# Assessment of Risk Factors Associated With Coronary Artery Disease in Riyadh, Saudi Arabia: A Cross-Sectional Study

**DOI:** 10.7759/cureus.84441

**Published:** 2025-05-19

**Authors:** Abdulelah Alshehri, Salahuddin Khan, Khalid Alshammari, Bandar Alotaibi, Faisal M Al-Mutairi, Rana Alhazzani, Osama Al-Ahmari, Meshal Alzakari

**Affiliations:** 1 College of Medicine, Imam Mohammad Ibn Saud Islamic University (IMSIU), Riyadh, SAU; 2 Department of Biochemistry, Imam Mohammad Ibn Saud Islamic University (IMSIU), Riyadh, SAU; 3 Department of Epidemiology, College of Health and Rehabilitation Sciences, Princess Nourah Bint Abdulrahman University, Riyadh, SAU

**Keywords:** cardiac risk factors and prevention, cardiology research, coronary artery disease, riyadh population, saudi arabia

## Abstract

Background and objective: Coronary artery disease (CAD) remains a leading cause of mortality worldwide. In Saudi Arabia, its prevalence is increasing due to modifiable risk factors such as obesity, diabetes, and hypertension. Raising public awareness of these factors is essential for effective prevention. This study aims to assess public knowledge and awareness of CAD risk factors and to examine their associations with demographic characteristics among adults in Riyadh, Saudi Arabia.

Methods: A cross-sectional study was conducted between January 21 and February 5, 2025, involving 901 participants aged 18 and older. A validated, structured questionnaire was distributed online to assess demographic characteristics and knowledge of CAD risk factors. Data were analyzed using RStudio (R Foundation for Statistical Computing, Vienna, Austria), with chi-square tests used to examine associations between demographic factors and CAD knowledge.

Results: Among the participants, 59.3% (n=534) were female, and 50.4% (n=454) were between 18 and 29 years of age. Overall, based on the scoring system, 88.1% (n=794) of participants demonstrated good awareness of CAD risk factors with a mean of 0.881 and SD (0.32), with smoking (n=697, 77.4%), physical inactivity (n=779, 86.5%), and obesity (n=855, 94.9%) being the most recognized. Higher awareness levels were significantly associated with female gender and postgraduate education (p < 0.05). However, gaps in knowledge persisted regarding diabetes and family history.

Conclusion: Although awareness of CAD risk factors is generally high, targeted educational initiatives are required to address misconceptions, particularly about non-modifiable risk factors. Strengthening public education efforts could help further reduce CAD prevalence in Saudi Arabia.

## Introduction

Coronary artery disease (CAD) remains a leading cause of mortality and morbidity worldwide, placing a significant burden on healthcare systems. Annually, CAD accounts for approximately 7.4 million deaths globally, with ischemic heart disease contributing substantially to mortality rates in both developed and developing nations. The prevalence of CAD varies across regions due to socioeconomic, demographic, and lifestyle factors, leading to disparities in disease burden and risk factor distribution [1,2].

While advances in healthcare and preventive strategies have helped stabilize CAD prevalence in developed countries, the burden continues to rise in developing regions, including the Middle East. This increase is primarily driven by rapid urbanization, changing lifestyle habits, and aging populations [3]. In Saudi Arabia, CAD-related morbidity has significantly increased, largely due to widespread risk factors such as obesity, diabetes, hypertension, and sedentary behaviors. Local studies estimate CAD prevalence at approximately 5.5% among individuals aged 30-70 years [4]; however, this figure may be underestimated due to the limited availability of large-scale, population-based studies For instance, Al-Nozha et al. reported a 5.5% prevalence based on a national survey [4], but subsequent studies have highlighted the scarcity of comprehensive, population-wide data, suggesting potential underestimation of true prevalence rates [5].

CAD risk factors are generally classified as modifiable or non-modifiable. Modifiable risk factors, including smoking, physical inactivity, hypertension, diabetes mellitus, obesity, and dyslipidemia, play a crucial role in disease progression and serve as key targets for preventive interventions [6,7]. Conversely, non-modifiable factors such as age, male gender, family history, and genetic predisposition also contribute significantly to CAD risk [8]. Research in Saudi Arabia and other Middle Eastern countries suggests that individuals often develop CAD at a younger age than global averages and frequently present with multiple coexisting risk factors [9].

Hypertension, dyslipidemia, and diabetes mellitus are particularly prevalent in the Middle East, with more than half of CAD patients in Saudi Arabia presenting with at least three risk factors. Insufficient awareness of the importance of managing these conditions further worsens disease outcomes. Additionally, the high prevalence of obesity and physical inactivity in the region compounds the public health challenges associated with CAD [10,11].

Effectively preventing and managing CAD requires a comprehensive, multifaceted approach. Awareness campaigns, early screening, and targeted interventions aimed at modifiable risk factors are crucial for reducing disease prevalence and improving health outcomes. Studies indicate that increased public awareness of CAD risk factors can promote healthier behaviors, encouraging preventive measures such as regular health screenings and lifestyle modifications [12]. However, research consistently highlights inadequate awareness levels among the Saudi population, underscoring the need for strengthened public health initiatives [13,14]. For example, a study in Dawadmi, Riyadh Province, revealed that a significant portion of participants lacked knowledge about CAD risk factors, emphasizing the necessity for targeted educational programs. [15].

This study aims to assess public knowledge and awareness of CAD risk factors and to examine their associations with demographic characteristics among adults in Riyadh, Saudi Arabia. Riyadh was selected due to its high population density, urbanization, and representativeness of the broader Saudi population, making it an ideal setting for understanding CAD risk factors and awareness levels [16]. By identifying gaps in knowledge and risk factor management, this research seeks to inform the development of targeted educational strategies to mitigate the rising burden of CAD in the region.

## Materials and methods

This was a cross-sectional study conducted among the general population in Riyadh, Saudi Arabia, between February 13 and February 25, 2025. The study received approval from the Institutional Review Board of Imam Mohammad bin Saud Islamic University (project number: 745/2024). Participants were fully informed about the study’s objectives, and their responses remained confidential, accessible only to the research team. Before participation, all individuals provided informed consent.

Eligibility criteria

The target population included adults aged 18 years and older, of both genders. To maintain objectivity, individuals working in the healthcare sector, such as physicians, pharmacists, and those with prior knowledge of coronary artery disease (CAD), were excluded. In this context, “prior knowledge” was defined as formal education or training in a medical or health-related field. A screening question was included at the beginning of the questionnaire to identify and exclude such individuals. 

Sampling and sample size

Convenience sampling was used to recruit participants via online platforms such as Instagram (Meta Platforms, Inc., Menlo Park, California, United States), LinkedIn Corporation (Sunnyvale, California, United States), WhatsApp (Meta Platforms, Inc.), Telegram (Telegram Group Inc., Dubai, United Arab Emirates), and Snapchat (Snap Inc., Santa Monica, California, United States) for practical and efficient reach. Participants met the inclusion criteria, ensuring diversity in gender, age, education, marital status, and employment. No quota or stratified sampling methods were applied, nor were statistical adjustments made after data collection.

Using the Raosoft sample size calculator (Raosoft Inc., Seattle, Washington, United States), the required sample size was estimated based on a 95% confidence level (CI) and a 5% margin of error, resulting in a minimum of 384 participants. To improve the accuracy and generalizability of the findings, the study ultimately included 901 participants.

Study tool

A validated questionnaire adapted from a previously published study [17] was used to assess CAD knowledge and awareness. Expert researchers translated the questionnaire into Arabic and structured it into three main sections (see Appendices). The first section collected demographic information, including gender, age, education level, marital status, and employment status. The second section evaluated participants’ understanding of CAD risk factors, while the final section included a consent form to ensure compliance with the university’s ethical standards for cross-sectional studies.

Data analysis

Data were collected online and processed using RStudio (R Foundation for Statistical Computing, Vienna, Austria). Descriptive statistics (frequency tables and graphs) were used to summarize the demographic characteristics and sources of CAD information. The chi-square test was employed to analyze associations between demographic variables and participants’ levels of CAD awareness. Adjusted residuals were used to further explore significant associations in gender, education, and employment status. The scoring system for awareness was based on 12 CAD-related questions. Each correct answer received one point, with total scores classified as follows: poor awareness (0-4), moderate awareness (5-8), and good awareness (9-12). Statistical significance was set at p < 0.05.

## Results

Among the 901 eligible participants, females constituted the majority (59.3%), and the most common age group was 18-29 years (50.4%). In terms of education, most respondents held a postgraduate degree (65.7%), followed by those with secondary education (29.9%). Regarding marital status, most participants were either single (50.3%) or married (45.1%). Students represented the largest employment group (40.4%) (Table 1).

**Table 1:** Sociodemographics characteristics of respondents (N=901)

Characteristics	Frequency	Percentage
Gender		
Female	534	59.3%
Male	367	40.7%
Age (years)		
18 - 29	454	50.4%
30 - 39	121	13.4%
40 - 49	204	22.6%
50 - 59	104	11.5%
60 and above	18	2.0%
Educational level		
Intermediate	30	3.3%
Primary	10	1.1%
Post graduate	592	65.7%
Secondary	269	29.9%
Marital Status		
Divorced	28	3.1%
Married	406	45.1%
Single	453	50.3%
Widowed	14	1.6%
Employment Status		
Employee	336	37.3%
Retired	65	7.2%
Student	364	40.4%
Unemployed	136	15.1%

**Figure 1:**
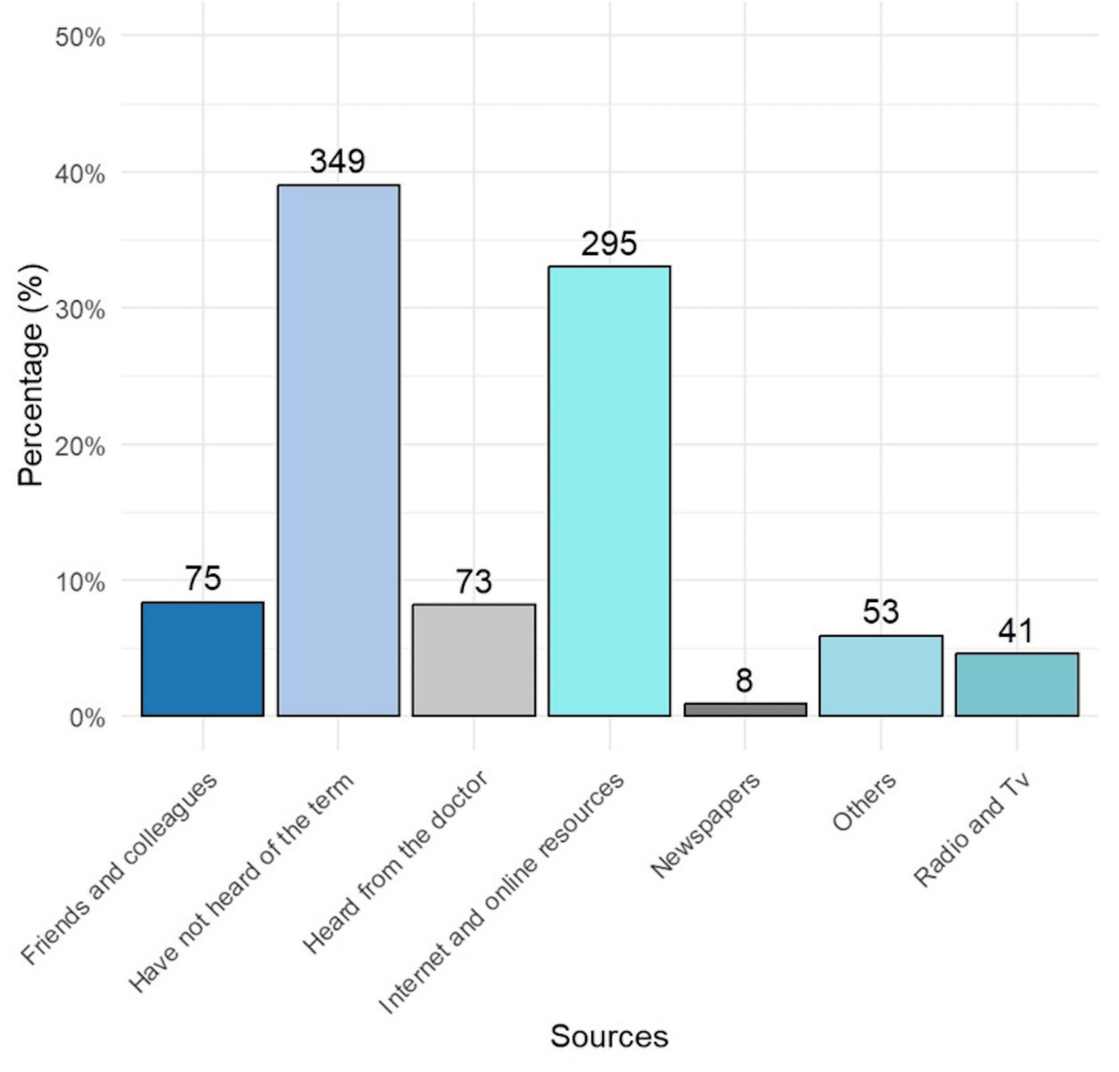
Source of information about coronary artery disease (N=901) Data on the bars are frequencies

Figure 1 illustrates the sources of information about CAD among respondents. A significant proportion (n=349, 39.0%) was unaware of the term "coronary artery disease". The internet and online resources were the most common sources of information, used by 295 respondents (33.0%), followed by friends and colleagues (75, 8.4%). Doctors provided information to 73 respondents (8.2%), while 53 (5.9%) cited other sources. Additionally, 41 respondents (4.6%) obtained information from radio and television, whereas newspapers were the least utilized source, mentioned by only eight participants (0.9%).

Table 2 summarizes the respondents’ knowledge of CAD and its risk factors. A large majority of participants acknowledged smoking (77.4%), lack of physical activity (86.5%), and poor dietary habits such as fast food (94.6%) and soft drinks (87.0%) as risk factors for CAD. Most respondents also recognized age (71.8%), family history (55.7%), high cholesterol (92.8%), high blood sugar (80.1%), obesity (94.9%), and stress (85.8%) as significant risk factors. There were divided opinions on gender differences, with 52.1% believing males are more susceptible to CAD. High blood pressure was recognized by 89.0% as a contributing factor.

**Table 2:** Summary of respondents’ knowledge about coronary artery disease (CAD) and its related risk factors (N=901)

Questions	Yes, n (%)	No, n (%)
Do you think that smokers are more likely to have cardiovascular disease?	697 (77.4%)	204 (22.6%)
Do you think that not exercising at least 30 minutes of walking daily for 5 days increases the incidence of cardiovascular disease?	779 (86.5%)	122 (13.5%)
Do you think that eating fast food increases the risk of cardiovascular disease?	852 (94.6%)	49 (5.4%)
Do you think that soft drinks increase the risk of cardiovascular disease?	784 (87.0%)	117 (13.0%)
Do you think that age is linked to cardiovascular disease?	647 (71.8%)	254 (28.2%)
Do you think that having a family member with cardiovascular disease increases your risk of cardiovascular disease?	502 (55.7%)	399 (44.3%)
Do you think that high cholesterol in the blood increases the risk of cardiovascular disease?	836 (92.8%)	65 (7.2%)
Do you think that high blood sugar (diabetes) increases the risk of cardiovascular disease?	722 (80.1%)	179 (19.9%)
Do you think that obesity increases the risk of cardiovascular disease?	855 (94.9%)	46 (5.1%)
Do you think that anxiety and stress increase the risk of cardiovascular disease?	773 (85.8%)	128 (14.2%)
Do you think that males are more susceptible to cardiovascular disease than females?	469 (52.1%)	432 (47.9%)
Do you think that high blood pressure increases the risk of cardiovascular disease?	802 (89.0%)	99 (11.0%)

**Figure 2:**
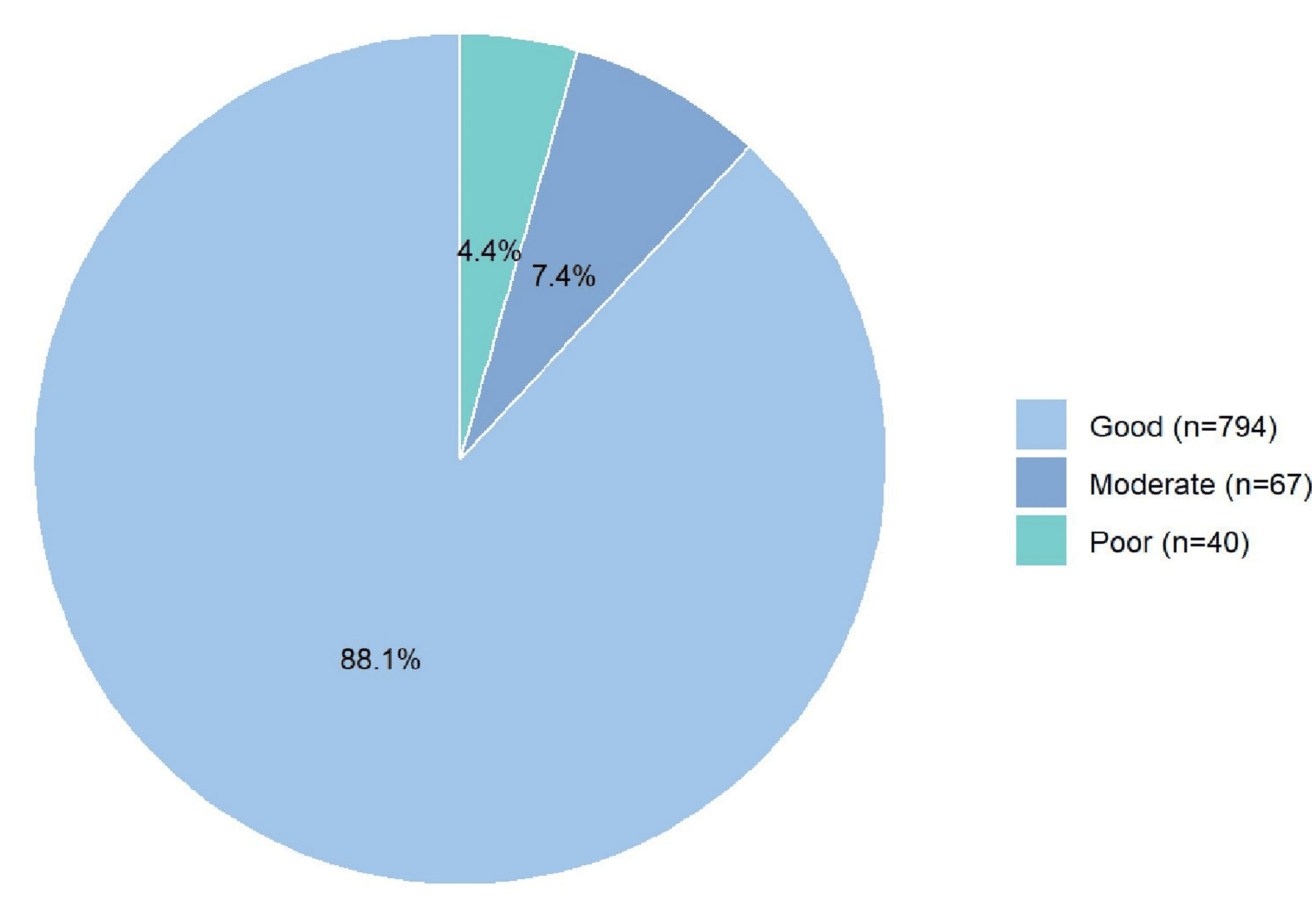
Level of awareness about the risk factors of coronary artery disease among all respondents (N=901)

Figure 2 illustrates that the majority of respondents (n=794, 88.1%) demonstrated good awareness of CAD risk factors. A smaller proportion exhibited moderate (n=67, 7.4%) or poor (n=40, 4.4%) awareness.

‏Table 3 shows significant associations between certain demographic characteristics and the level of awareness about CAD. Gender was significantly associated with awareness levels (p = 0.010), with a higher proportion of females (90.8%) demonstrating good awareness compared to males (84.2%). Conversely, males had higher proportions of moderate (9.8%) and poor awareness (6.0%) than females (5.8% and 3.4%, respectively). Educational level also showed a significant association (p = 0.002). Participants with a postgraduate degree had the highest rate of good awareness (90.5%), while those with intermediate education had the lowest (80.0%). Notably, 16.7% of respondents with intermediate education reported poor awareness, the highest among all education levels. Older participants tended to score higher. For instance, 94.4% of respondents aged 60 and above demonstrated good awareness, compared to 84.8% of those aged 18-29. Employment status was significantly associated with CAD awareness (p = 0.025). Retirees (93.9%) and employees (91.1%) had the highest rates of good awareness, while students had the lowest (84.3%). Poor awareness was particularly low among unemployed participants (2.9%). Marital status, despite the small sample of widowed respondents, all demonstrated good awareness, while single participants had slightly higher rates of moderate (8.6%) and poor (5.3%) awareness compared to other groups.

**Table 3:** The association between respondents' characteristics and their knowledge of coronary artery disease (N=901) p-value < 0.05 was considered significant Data given as mean (SD) and frequency (row percentage)

Characteristics	Good (n=794)	Moderate (n=67)	Poor (n=40)	p-value	Pearson chi^2^
Gender, mean (SD)	1.389 (0.487)	1.537 (0.51)	1.55 (0.50)	0.010	9.1466
Female, n (%)	485 (90.82%)	31 (5.81%)	18 (3.37%)		
Male, n (%)	309 (84.20%)	36 (9.81%)	22 (5.99%)		
Age, mean (SD)	2.057 (1.18)	1.701 (1.05)	1.65 (1.00)	0.209	10.8715
18-29, n (%)	385 (84.80%)	43 (9.47%)	26 (5.73%)		
30-39, n (%)	109 (90.08%)	7 (5.97%)	5 (4.13%)		
40-49, n (%)	186 (91.18%)	12 (5.88%)	6 (2.94%)		
50-59, n (%)	97 (93.27%)	4 (3.85%)	3 (2.88%)		
60 and above, n (%)	17 (94.44%)	1 (5.56%)	0 (0.00%)		
Educational level, mean (SD)	3.211 (0.61)	3.358 (0.59)	3.175 (0.95)	0.002	20.5709
Intermediate, n (%)	24 (80.00%)	1 (3.33%)	5 (16.67%)		
Primary, n (%)	9 (90.00%)	1 (10.00%)	0 (0.00%)		
Postgraduate, n (%)	536 (90.54%)	38 (6.42%)	18 (3.04%)		
Secondary, n (%)	225 (83.64%)	27 (10.04%)	17 (6.32%)		
Marital Status, mean (SD)	2.494 (0.59)	2.567 (0.52)	2.55 (0.59)	0.412	6.0981
Divorced, n (%)	25 (89.29%)	1 (3.57%)	2 (7.14%)		
Married, n (%)	365 (89.90%)	27 (6.65%)	14 (3.45%)		
Single, n (%)	390 (86.09%)	39 (8.61%)	24 (5.30%)		
Widowed, n (%)	14 (100%)	0 (0.00%)	0 (0.00%)		
Employment Status, mean (SD)	2.303 (1.13)	2.522 (1.10)	2.6 (0.98)	0.025	14.4120
Employee, n (%)	306 (91.07%)	20 (5.95%)	10 (2.98%)		
Retired, n (%)	61 (93.85%)	4 (6.15%)	0 (0.00%)		
Student, n (%)	307 (84.34%)	31 (8.52%)	26 (7.14%)		
Unemployed, n (%)	120 (88.24%)	12 (8.82%)	4 (2.94%)		

## Discussion

This study provides a comprehensive evaluation of risk factors associated with CAD and assesses public awareness levels in Riyadh, Saudi Arabia. A substantial proportion of participants (88.1%) demonstrated a strong understanding of CAD risk factors, while 7.4% exhibited moderate awareness and 4.4% displayed poor awareness. Notably, significant associations emerged between awareness levels and demographic characteristics, including gender, educational attainment, and employment status. Female participants and those with postgraduate education exhibited higher awareness scores, suggesting that education plays a pivotal role in shaping knowledge about cardiovascular health.

A comparison with previous studies highlights both consistencies and disparities. Ammouri et al. reported that only 60% of their participants achieved adequate knowledge scores regarding CAD risk factors [18], whereas the present study indicates a notably higher awareness level. This discrepancy may reflect the impact of recent public health campaigns and increased digital health literacy in Riyadh. Similarly, Awad and Al-Nafisi found that smoking, obesity, and physical inactivity were widely recognized as major risk factors [14]; this aligns with our findings, where over 90% of respondents identified smoking as a risk factor and more than 88% acknowledged the risks associated with high cholesterol and hypertension. Conversely, Wartak et al. observed that individuals with prior cardiac events did not necessarily possess greater knowledge about CAD compared with the general population [19]. The results of the current study support this notion, demonstrating that awareness levels remain high across the general population, likely due to widespread preventive education efforts. However, Almalki et al. reported moderate awareness levels in Jeddah, with lower recognition of risk factors such as diabetes and family history [20]. The current study identified similar gaps, reinforcing the need for targeted educational initiatives that emphasize these overlooked risk factors. It is worth noting that these studies differed in setting, population demographics, and data collection periods, which may account for variations in reported awareness levels. Our findings should therefore be interpreted within the context of these methodological and geographical differences.

These findings underscore the effectiveness of existing public health efforts in promoting awareness of key modifiable risk factors, such as smoking, obesity, and hypertension. However, gaps remain in public knowledge, particularly regarding diabetes and family history, which suggests that current health education strategies may not sufficiently emphasize these risk factors. Tailored interventions should prioritize populations with lower awareness levels, especially individuals with limited educational backgrounds. Furthermore, the high awareness observed in this study, particularly among younger and well-educated participants, suggests that internet-based health campaigns may contribute to increased awareness; however, this remains a hypothesis, as the current study did not directly assess the impact of such campaigns.

Strengths of the study

Our study offers several strengths that contribute to its relevance and impact. It involved a relatively large and diverse sample of 901 participants from Riyadh, enhancing the statistical power and the reliability of the findings. The use of a validated, structured questionnaire ensured consistency in data collection. Furthermore, the study explored associations between awareness levels and key demographic variables, providing insights into patterns of knowledge distribution across population subgroups. One particularly noteworthy finding is the reliance on the internet as the primary source of information about coronary artery disease (33%), compared to only 8.2% who cited healthcare professionals. This highlights both the growing role of digital platforms in public health education and the potential risks of misinformation in the absence of professional guidance, and underscores the need to optimize digital health strategies and strengthen the integration of healthcare providers in preventive health communication.

Limitations of the study

Despite the strengths, the study has several limitations. While our results indicate a generally high level of awareness, these findings should be interpreted with caution. Its cross-sectional design prevents any causal inferences between demographic factors and awareness levels.. The use of a convenience sample distributed online may have resulted in selection bias, potentially overrepresenting individuals with higher health literacy and educational attainment. As such, the generalizability of our results to the broader Riyadh population is limited. We rephrased the interpretation of internet-based campaigns as a hypothesis rather than a conclusion, and contextual differences were noted when comparing to other studies. Additionally, non-significant findings were addressed, and possible cultural factors, including gender roles and health information access, were discussed as potential contributors to the observed disparities.

The use of a self-administered online questionnaire may have introduced selection bias, favoring younger and more health-conscious individuals with internet access, as well as recall bias. The high proportion of participants with postgraduate education suggests a sampling skew, which may have inflated overall awareness estimates and limited representativeness. Furthermore, limiting the study to a single city, Riyadh, reduces the generalizability of the findings to the broader Saudi population. Employment status was assessed through mutually exclusive categories; however, potential overlap between roles such as “student” and “employee” may have introduced classification ambiguity. Lastly, while the study identified significant associations between awareness and certain demographics, other non-significant variables warrant further exploration. Future studies should adopt randomized, multicenter designs and consider longitudinal approaches to better assess trends over time and evaluate the effectiveness of targeted awareness interventions.

## Conclusions

This study reveals a generally high awareness of CAD risk factors among residents of Riyadh, particularly among females and those with higher educational attainment. However, the use of an online survey may limit the representativeness of the findings, introducing potential bias toward more educated and internet-active individuals. Thus, the generalizability to the entire Riyadh population should be interpreted with caution. Moreover, the cross-sectional nature of the study precludes the assessment of long-term knowledge retention or behavioral impact, underscoring the need for longitudinal or follow-up studies.

Notably, certain groups, such as students, males, and individuals with intermediate education, demonstrated lower levels of awareness, suggesting the need for targeted interventions. Public health efforts should focus on reaching these populations through tailored approaches. Suggested strategies include school-based programs for students, workplace wellness initiatives for males, and community-driven awareness campaigns for less-educated groups. Digital campaigns, while widely accessed, should be supplemented with professional healthcare guidance to ensure accuracy and efficacy.
